# The history of brucellosis in the Middle East: insights for contemporary health challenges

**DOI:** 10.3389/fmicb.2025.1571087

**Published:** 2025-05-30

**Authors:** Maryam Dadar, Robin Bendrey, G. Michael Taylor, Youcef Shahali

**Affiliations:** ^1^Department of Brucellosis, Razi Vaccine and Serum Research Institute (RVSRI), Agricultural Research, Education and Extension Organization (AREEO), Karaj, Iran; ^2^School of History, Classics and Archeology, University of Edinburgh, Edinburgh, United Kingdom; ^3^School of Biosciences, University of Surrey, Guildford, United Kingdom; ^4^Centre Hospitalier Universitaire de Besançon, Besançon, France

**Keywords:** *Brucella*, one health, archeology, public health education, policy *Brucella*, policy

## Abstract

Brucellosis, a significant zoonotic disease in the Middle East, presents substantial economic and public health consequences. Despite concerted efforts toward its control and eradication, managing the disease faces various challenges. While One Health approaches prioritize interdisciplinary actions to enhance disease understanding, communication, and policy, they often overlook historical perspectives. These regions experience significant brucellosis loads attributed to livestock density, pastoralism, and transboundary animal migration. Historical documentation, encompassing old medical manuscripts, indicates potential early hints of brucellosis-like diseases. The primary *Brucella* species circulating in the Middle East include *Brucella abortus*, *Brucella melitensis*, and *Brucella suis*, with *B. melitensis* most commonly associated with human brucellosis. Political and social instability, insufficient finances for disease control programs, and gaps in diagnostic and monitoring infrastructure all provide difficulties for Middle Eastern governments trying to control brucellosis. Although vaccination strategies have been successful, logistical issues have caused inconsistent application. Reducing the spread of the disease depends critically on extra-preventative actions, including public awareness campaigns, controlled cattle trading, and biosecurity enforcement. This study underscores the significance of exploring the rich historical evidence and knowledge of brucellosis, emphasizing its potential contributions to public education, policy development, and improved health outcomes.

## Introduction

**FIGURE 1:**
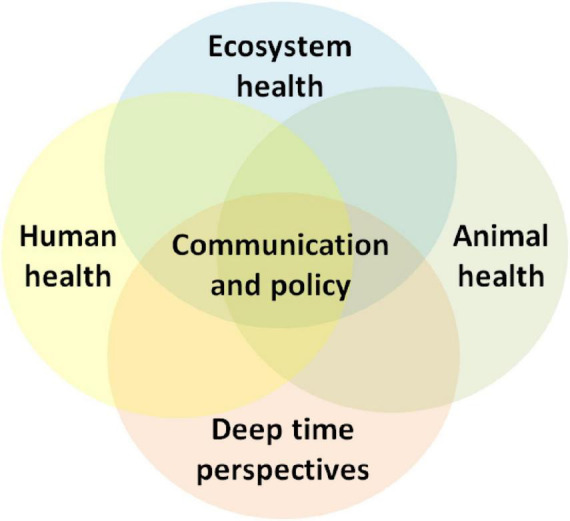
The One Health approach acknowledges and promotes the health of interconnected socio-ecological systems, which are best understood from deep time perspectives (Bendrey and Fournié, 2023; Rayfield et al., 2023). Effective policy implementation requires meaningful engagement with health messaging and recognition of community livelihoods as integral components of these systems.

Brucellosis is a common zoonotic disease of global significance, affecting animals and humans with significant health and economic impacts. In the majority of Middle Eastern countries, human and animal brucellosis remains endemic, accounting for high numbers of new annual cases (Musallam et al., 2016). The annual global human cases of brucellosis are approximately 2.1 million (Laine et al., 2023). Despite considerable efforts and partial successes, there are still several obstacles in the region that health authorities need to overcome to effectively control and eradicate the disease (Dadar et al., 2021). A One Health approach offers the most potent framework for developing and delivering effective and successful control programs (Godfroid et al., 2013; Dadar et al., 2024). This attempt to implement holistic and interdisciplinary collaborations to improve disease knowledge, communication, and policy (e.g., Figure 1). To date, such approaches have not effectively drawn upon deep-time perspectives (Bendrey and Fournié, 2021) but rather consider socio-ecological disease dynamics from a temporally shallow perspective. Here, we wish to highlight longer-term perspectives that value insights that understanding long-term biological, ecological, and cultural evolution can provide. This includes the contextualization of long-term changes in the ecology of these diseases and the development in our understanding of the adaptive and interactive capacity of this widely spread infectious agent. Long-term changes in perceptions and scientific understanding of diseases can help identify past, present, and future drivers. They may provide new outcomes for private actions and public health policy at regional and global levels.

**FIGURE 2:**
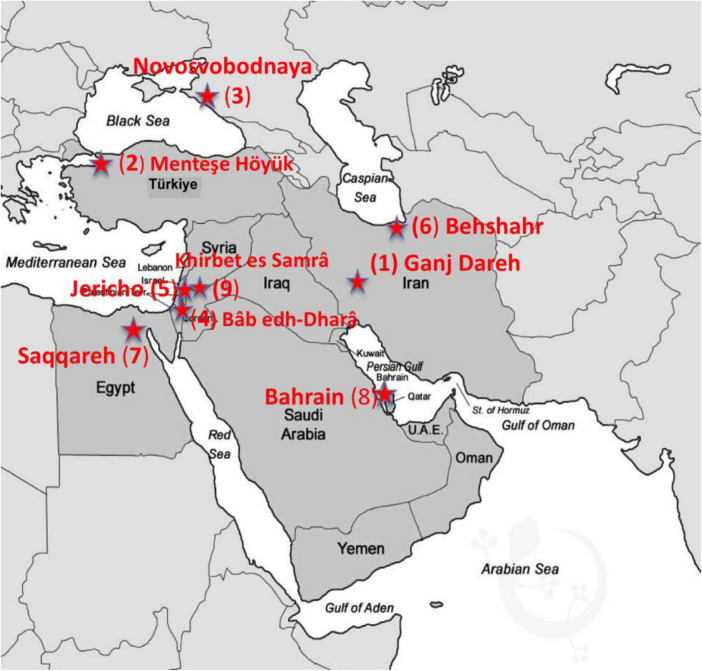
Map of the Middle Eastern countries with archeological sites (red stars) where evidence of brucellosis has been recovered from human and animal remains via DNA or paleopathology (The numbers on the map correspond to the data presented in Table 1 under the “Map Star” column).

This contribution considers the current situation relating to brucellosis in the Middle East. Middle Eastern countries include Lebanon, Cyprus, Iran, Syria, Iraq, Israel, Saudi Arabia, Jordan, Qatar, Kuwait, United Arab Emirates, Bahrain, Yemen, Egypt, Palestine, Türkiye, and Oman (Alsulami et al., 2013). We then consider knowledge and evidence of the disease moving back through the era of microbiology, history, and then archeology (Figure 2). In reviewing archeological and historical evidence for the disease in this region (e.g., Table 1), we aim to consider what these long-term contexts can contribute to addressing current challenges.

**TABLE 1:** Selected summary archeological and historical evidence for both definitive and possible brucellosis from the Middle East and the surrounding areas.

Time period	Location	Evidence type and notes	Reference	Map star
End 9th–8th millennium BCE	Zagros mountains of western Iran and eastern Iraq	Simulation modeling of the transmission of *Brucella melitensis* in early domestic goat populations described by zoo archaeological data indicates potential for endemicity in the early phases of animal husbandry.	Fournié et al., 2017	=
	Ganj Dareh, western Iran	Paleopathological evidence for a possible case of Early Neolithic brucellosis in an adult human skeleton in an early goat husbandry context	Merrett, 2002; Merrett, 2004	1
Late 7th/early 6th millennium BCE	Menteşe Höyük, Northwest Türkiye	3.45X *Brucella melitensis* genome recovered from a Neolithic sheep petrous bone	L’Hôte et al., 2024	2
4th millennium BCE	Novosvobodnaya, North Caucasus	*Brucella abortus* DNA recovered from an Early Bronze Age human skeleton	Sokolov et al., 2016	3
4th-3rd millennium BCE	Bâb edh-Dhrâ, Jordan	Bronze Age human paleopathological evidence suggestive of brucellosis	Ortner and Frohlich, 2007	4
3rd millennium BCE	Jericho, Palestine	Bronze Age human paleopathological evidence suggestive of brucellosis	Brothwell, 1965	5
Late 3rd millennium BCE	The Akkadian Empire (an area from the Persian Gulf to the Mediterranean Sea)	Indirect textual evidence (Akkadian) describing a disease called *maškadum* in animals interpreted as possibly brucellosis	Wasserman, 2012	=
2nd millennium BCE	Gohar Tepe, Behshahr, Northern Iran	Reported presence of *Brucella* aDNA evidence in Bronze Age human and animal dental samples	Kafil et al., 2014	6
Late 2nd millennium BCE	Saqqara, tomb of Ptahmes, Egypt	Proteomic analysis of cheese residues. The peptide sequence described as indicative of *B. melitensis* (GSIKER) could conceivably have originated from another organism, *Coxiella burnetii*, a Gram-negative organism affecting ruminants.	Greco et al., 2018	7
Bronze Age 2,000-1,800 BCE	Bahrain	Bronze Age human paleopathological evidence suggestive of brucellosis.	Rashidi et al., 2001	8
Mid 1st millennium BCE	Greece	Textual evidence—Hippocrates describes symptoms similar to brucellosis	Pappas et al., 2008	=
7th–9th century CE	Khirbet es-Samra, Jordan	Paleopathological signs on two adult male skeletons indicative of brucellosis	Nabulsi et al., 2020	=
12th century CE	Iran	Persian physician Jorjani describes “hectic fever” in his medical text “Zakhireh kharazmshahi,” a disease with symptoms resembling those of brucellosis, although also shares symptoms with other fevers, so is not definitive evidence of the disease.	Tadjbakhsh, 2007	=

### Methodology for the synthesis and selection of historical and contemporary sources

We selected historical, genetic, archeological, and epidemiological data to comprehensively reveal an interdisciplinary analysis of brucellosis in the Middle East. With careful attention to well-documented records in peer-reviewed journals and academic books, we searched for evidence of historic brucellosis in ancient animals and human populations using an exhaustive literature review including archeological and paleopathological investigations. From this review, verified ancient DNA analyses and osteoarchaeological findings were chosen for geographical and chronological studies, giving data showing evidence of brucellosis in the Middle East from the Neolithic through to the early modern period as the first priority. By means of a systematic review of Whole Genome Sequencing (WGS) studies and phylogenetic analysis of *Brucella* species circulating in the region, we selected studies according to the application of genetic markers and strain discrimination techniques such as WGS, multi-locus sequence typing (MLST), and single nucleotide polymorphism (SNP) analysis, so linking ancient and modern cases and assessing phylogenetic relationships and continuity over time. Considering endemicity, risk factors, and transmission pathways for human and animal brucellosis in the Middle East, epidemiological data were pooled from regional and worldwide health organizations and peer-reviewed literature. Long-term brucellosis transmission could be inferred by combining the epidemiological patterns with historical and molecular data. To link past epidemics with contemporary illness patterns, we finally combined historical, molecular, and epidemiological data inside a comparative framework using qualitative and quantitative techniques including phylogenetic analysis and spatial-temporal mapping. These data were carefully examined for congruence between ancient and present findings, therefore providing a more complex picture of *Brucella’s* evolution and consequences for current public health campaigns.

### Brucellosis and its impacts in the Middle East today

#### The pathogen and its health impacts

Brucellosis is an infection caused by Gram-negative intracellular bacteria of the genus *Brucella* (Gwida et al., 2010; Addis, 2015). Although it predominantly impacts domestic livestock such as cattle, goats, sheep, pigs, and wildlife, as a zoonotic disease, it can also be transmitted to humans. It has been eradicated in some developed countries, but it remains a divesting zoonotic disease in many developing countries, seriously affecting farming areas in the countries of the Middle East (Brangsch et al., 2023; El-Diasty et al., 2021; Ilyas et al., 2024).

Brucellosis encompasses various species that have the capability to infect people. These species include *Brucella abortus*, *Brucella melitensis*, *Brucella suis*, and *Brucella canis* (Moreno, 2014). These bacteria are transmitted to humans by direct or indirect contact with diseased animals or their by-products, including unpasteurized milk, meat, and other dairy products (Doganay and Aygen, 2003). Brucellosis can manifest in humans with various symptoms, including fever, sweats, weakness, muscle and joint pain, and general malaise. The disease can become chronic and cause more severe complications if left untreated, affecting multiple organs and systems, including the heart, liver, spleen, and bones (Dadar et al., 2019). It is important to note that brucellosis is a significant concern for public health and the livestock industry, as it can result in economic losses due to decreased livestock productivity.

The disease has been assigned more than 50 names throughout history until it was eventually acknowledged as a unified entity. This highlights the diverse and complex nature of the disease, which has shown various complexities and manifestations. Consequently, these characteristics have given rise to puzzling issues that have persisted or reappeared after their initial documentation (Moreno et al., 2022). As initially documented by Hughes (1897), the incubation period of human infection exhibits variability and can sometimes be prolonged. During this period, individuals may have a range of non-specific symptoms and signs that are not unique to the infection, leading to frequent misdiagnosis with other illnesses and in some cases, even resulting in fatality (Grattarola et al., 2023), Surgeon-Captain David Bruce, in his influential study published in 1887 (Bruce, 1887) documented the identification of *Brucella melitensis* (originally assigned as *Micrococcus melitensis*). Bruce observed that the microscopic characteristics of the spleen, kidney, and liver closely resembled those observed in diseases caused by microorganisms, such as enteric fever and scarlet fever (Miller et al., 1999). Significantly, within certain species, such as dolphins, this disease exhibits a notable impact on both the central neurological and reproductive systems, resulting in occurrences of abortions and mortality (Miller et al., 1999; Dadar et al., 2022; Grattarola et al., 2023). On the other hand, the infection in sheep, cattle, pigs, and goats typically remains asymptomatic for much of the time, with noticeable manifestations such as abortions and heightened neonatal mortality only occurring toward the latter stages of pregnancy. Hence, it is unsurprising that, over a span of more than thirteen decades since the identification of *B. melitensis* and 100 years since the characterization of the *Brucella* genus (Miller et al., 1999; Dabney et al., 2013), the diagnosis, identification, treatment, control, and prevention of the disease in both animals and humans continue to provide a challenge. In the year 1912, a short time prior to Alice Evans’ discovery of the connection between *B. abortus* and *B. melitensis*, which impact cattle and small ruminants, respectively, the US Veterinary and Public Health services offered evidence regarding the association between human and animal infections resulting from the ingestion of unpasteurized dairy products. The disease is still considered as a neglected zoonotic disease because of the restricted resources devoted to its control and surveillance, especially in developing countries (Arimi et al., 2005; McDermott et al., 2013). Although *Brucella* infection in humans is rarely fatal, it is more severe than in livestock and remains a significant concern for public health through the different clinical signs and symptoms (Franco et al., 2007). Human brucellosis is strongly connected to ingesting unpasteurized dairy products and managing infected animals (Dadar et al., 2019). Therefore, there is a close link between human brucellosis and the adoption of farming, milking practices, and the production of dairy products. The term “undulant fever” was coined in the nineteenth century CE to describe the recurring fever pattern associated with brucellosis. This nomenclature illustrates the recognition of the disease’s cyclical nature (Miller et al., 1999). More contemporary records, such as clinical case reports and medical literature from the twentieth century, have significantly improved our understanding of brucellosis, its symptoms, and its transmission (Young, 1995).

#### Economic impacts

This zoonotic disease has significant economic implications, as it affects agriculture, livestock production, and human health, leading to direct and indirect costs for affected nations and communities. The financial effects on agriculture and livestock production are of significant importance. The occurrence of brucellosis in cattle has been seen to have a negative impact on productivity, specifically in terms of reduced milk and meat production. Additionally, infected animals have been found to exhibit lower fertility rates, which subsequently leads to a drop in breeding success (McDermott and Arimi, 2002; McDermott et al., 2013). This can be particularly relevant where calves form a significant source of income. The economic consequences on public health encompass the financial burdens associated with healthcare expenses, such as the costs incurred for diagnosing, treating, and hospitalizing individuals affected by brucellosis. These expenditures have a substantial impact on healthcare budgets. Moreover, brucellosis results in decreased work productivity as individuals affected by this disease commonly endure lengthy periods of illness, resulting in increased absenteeism and diminished productivity. Ultimately, this disease can result in chronic complications such as osteoarticular and neurological problems, so exerting a long-term adverse impact on the overall quality of life (Herrick et al., 2014). The financial stresses of overburdened healthcare facilities and the necessity for diagnostic infrastructure in healthcare systems, as well as monitoring and controlling brucellosis, could have other economic impacts (Moreno et al., 2023). Brucellosis can, therefore, cause high economic losses and health impacts. The most critical risk factors facilitating the spread of brucellosis are: (1) the complexity of, and the difficulty in maintaining, strict control programs which in turn can be highly impacted by political and social instabilities; (2) systems of livestock husbandry and mixed farming involving other livestock and other wildlife animals; (3) insufficient resources for implementation of control measures; (4) suitable diagnosis and reporting; and (5) traditional cultural practices such as the consumption of unpasteurized dairy products (Musallam et al., 2016; Dadar et al., 2024).

#### Contemporary health challenges in the Middle East

Recent research shows that brucellosis is a significant public health risk in the Middle East and several parts of North Africa (Holzer et al., 2022; Akar et al., 2024). The issue of underreporting is a significant problem within the current health challenges related to brucellosis in the Middle East. This obstacle arises from various factors, including inadequate awareness, limited healthcare accessibility, and misclassifying brucellosis cases as alternative febrile illnesses (Dadar et al., 2024). The gathering and reporting of precise and reliable data are essential in order to tackle these matters effectively. Livestock management is another problem due to the close interaction between humans and livestock in the Middle East, particularly in rural areas, which contributes to the spread of brucellosis. Inadequate veterinary care, poor vaccination coverage, and suboptimal livestock management practices pose ongoing challenges (Dadar et al., 2021). Furthermore, weak borders and the unrestricted movement of animals within the region provide significant obstacles in effectively managing brucellosis transmission. The proper resolution of this situation necessitates regional cooperation (Bagheri Nejad et al., 2020).

In the Middle East, brucellosis significantly affects food animal production and public health (Pappas and Memish, 2007). About 5% of the world’s population and land area, along with 6% of the world’s sheep, goats, and cattle, are located in these countries (Bagheri Nejad et al., 2020). The density of goats and sheep in this region is about twice as high as those reported in other parts of the world, contributing toward increased close contact with animals, increased rates of mixed farming, and increased burden of human brucellosis. The seroprevalence values of small ruminant populations in the region rank among the highest globally (Musallam et al., 2016). The common practice of pastoralism in Middle Eastern countries and the consumption of unpasteurized dairy products by traditional customs are important risk factors facilitating *Brucella* transmission from animal to human. In these areas, the shedding of the bacteria in milk and reproductive discharges of bovines and small ruminants are the primary sources of *Brucella* infection for humans and other animals (Hotez et al., 2012). The prevalence of *Brucella* spp. in dairy products in the Middle East region was estimated to be 29% (Abedi et al., 2020). Moreover, available studies reported an extensive seroprevalence and presence of human brucellosis in this region based on serological surveys at local levels (Pappas and Memish, 2007; Musallam et al., 2016). The main species causing human brucellosis in the Middle East, where people economically depend on ruminant livestock, are *B. abortus*, *B. melitensis*, and *B. suis* (Musallam et al., 2016). Other available data commonly show that small ruminant brucellosis is most prevalent, and *B. melitensis* is the most frequently identified cause of human brucellosis (Banai et al., 2018). To address the problem of brucellosis in the Middle East, countries are implementing control measures such as vaccination, which have been proven effective (Dadar et al., 2021; Bahmani and Bahmani, 2022). Other preventive measures include improved sanitary measures in livestock production and consumption, enhanced biosecurity, controlled animal sale, quarantine, and enhanced monitoring of the disease in humans and animals. It is also essential to increase public awareness of the zoonotic possibility of brucellosis and to strengthen the implementation of diagnostic protocols and management strategies for humans and animals.

#### *Brucella* and the era of microbiology

*Brucella* as a pathogen became known to medicine in Malta in the late nineteenth century, as outlined above (Bruce, 1887; Hughes, 1897), triggering the beginnings of international recognition of this disease. In Türkiye, for example, *Brucella* organisms were first isolated from a human subject (a soldier) in 1905 (Mackie, 1933) and from an animal (cattle) in 1931-2. Similarly, in Iran, *B. melitensis* was first isolated from human blood culture in 1932, and in 1944, *B. abortus* was isolated from an aborted cattle fetus (Esmaeili, 2014)

The organisms of the *Brucella* genus have been acknowledged as one of the first identified zoonotic bacteria, therefore attaining a prominent status in the annals of infectious diseases. Nevertheless, due to the absence of prominent characteristics during cultivation and the lack of definitive signs and symptoms in the disease they induce (brucellosis), it has required more than a century to recognize the striking similarity between strains isolated from various hosts and to establish the classification of the *Brucella* genus (Moreno, 2021). In 1975, taxonomic criteria were applied that emphasized the inclusion of species in the *Brucella* genus based on their intracellular pathogenicity and virulence features, thus setting minimum standards for classification (Corbel and Brinley Morgan, 1975). Approximately 10 years later, a team of researchers demonstrated that these pathogens exhibited a close relationship with some soil bacteria that sporadically gave rise to opportunistic nosocomial infections (De Ley et al., 1987). This cluster was ultimately identified as the *Ochrobactrum* genus (Corbel and Brinley Morgan, 1975). It was subsequently realized that *Ochrobactrum* and *Brucella* belong to Class 2 Alphaproteobacteria, exhibiting close phylogenetic relationships with plant pathogens and endosymbionts like Sinorhizobium and Agrobacterium (De Ley et al., 1987). Both genera fall under the order Rhizobiales (syn. Aphomicrobiales), which is a taxonomical grouping encompassing the family Brucellaceae, and a closely related family, Bartonellaceae (Blasco et al., 2021). Historically, the *Brucella* and *Ochrobactrum* species have been classified into separate genera due to notable morphological, genotypic, biochemical, and epidemiological distinctions (Moreno et al., 2023; Holzer et al., 2023). *Brucella* has a smaller genome (3.1–3.4 Mb) and lacks plasmids, while *Ochrobactrum* has a larger genome (4.7–8.3 Mb) and possesses multiple plasmids (Moreno et al., 2023).

#### Early written records—literary and historical insights

Early written records can also provide valuable insights into a disease’s historical presence and impact. While direct mentions of brucellosis in ancient texts are rare, some described conditions can provide indirect evidence for the disease. However, interpretations should consider differential diagnoses for identified symptoms (see Table 1 for selected examples). The genetic diversity observed in ancient samples aligns with modern strains circulating in countries along the Silk Road and the broader Middle East. Shared genotypes among strains from Türkiye, China, Kazakhstan, and Egypt suggest historical introduction and reintroduction through trade and animal movement (Liu et al., 2021).

The historical understanding of brucellosis is often traced back to Hippocrates, the ancient Greek physician (born in 460 BCE), who described symptoms consistent with this disease in the 7th part of the Aphorisms (vignette 64) (Pappas et al., 2008). This is one of the earliest recorded references to a brucellosis-like illness, although the causative agent was unknown then. Hippocrates refers to the coexistence of “tumors” and joint pain in cases of prolonged fevers, a correlation that could potentially align with the manifestation of the illness (Capasso, 2002; Ortner and Frohlich, 2007). Staying in the eastern Mediterranean, Kousoulis et al. (2012) critically analyze the text of Oedipus Rex by Sophocles around 430-420 BCE and suggest a possible diagnosis of *Brucella abortus* for the epidemic described.

There are earlier possible references to brucellosis, although like with many disease descriptions before modern medical knowledge, diagnoses are uncertain and may conflate different diseases, and consequently, there is debate about their identification (Wasserman, 2012). Akkadian sources describe a disease called *maškadum* in animals, interpreted as possible brucellosis by Wasserman (2012). The text highlights that *maškadum* affects humans and animals, mainly milking cows. It presents symptoms such as severe muscle and joint pain, often described as a “wolf bite” or “scorpion’s/snake’s venom.” Later sources emphasize its impact on the hips, shins, ankles, loins, back, and heel tendons, affecting the entire body. These details strengthen the argument for linking *maškadum* with brucellosis, a zoonotic disease known to impact both livestock and humans.

Further indirect evidence may also support awareness of the diseases in later historic periods, although descriptions are non-specific and may refer to other illnesses. Tadjbakhsh (2007), for example, writes that the 12th century CE Persian physician Jorjani describes “hectic fever” in his medical text “Zakhireh kharazmshahi,” a disease resembling symptoms of brucellosis, although also sharing symptoms with other fevers, so is not definitive evidence of the disease (Tadjbakhsh, 2007). In the Mediterranean region, Malta fever was well-documented in the nineteenth century CE, and historical medical texts provide insights into its symptoms and prevalence (Grattarola et al., 2023).

#### Improved simulation modeling and genomic evidence for *Brucella* transmission and control

Simulation modeling integrates ecological, socio-economic, and epidemiological factors to simulate disease spread under different conditions. This is essential for evaluating control measures and analyzing *Brucella* transmission dynamics. These models could forecast potential outbreaks and assess the effectiveness of vaccination programs, culling, and biosecurity measures by examining data on animal reservoirs, human contact, environmental survival, and control strategies. Models often categorize populations into compartments, including susceptible, infected, and recovered (SIR) individuals, for both humans and livestock (e.g., sheep, goats, and cattle). Prevalence data and historical records help to modify transmission rates between species and populations (Anyanwu et al., 2024; Sun et al., 2020). Mathematical models can investigate complex epidemiological characteristics of infectious diseases and transmission paths, thereby implying fresh approaches for controlling and preventing the next epidemics (Sun et al., 2020). Using ordinary differential equations for diverse circumstances, dynamic brucellosis transmission models in both single and multiple populations are created. Alternative modeling approaches, such as agent-based models (ABMs), capture behavior, movement, and disease transmission heterogeneity within specific environments. Compartmental models, for instance, SIR, which describes the population in three compartments depending on infection status, have been employed to model transmission pathways among livestock and human populations. These models are especially effective in characterizing localized epidemics and assessing the effects of treatments at a micro-level (Nie et al., 2021). Policymakers employ these models to formulate evidence-based interventions, enhance immunization efforts, and broaden monitoring operations to mitigate disease burden. Furthermore, globalization and climate change influencing *Brucella* transmission will enable the models to guide future risk assessments and strengthen early warning systems in endemic areas.

Genomic investigations provide essential insights into the evolution of *Brucella*, host adaptation, and transmission pathways. Recent genomic studies have discovered unique *Brucella* lineages in the Middle East, demonstrating regional transmission patterns and transnational spread (Holzer et al., 2022; Akar et al., 2024). Other studies have also revealed genomic differences between modern and ancient strains, underscoring *Brucella*’s longstanding presence and evolutionary adaptations (L’Hôte et al., 2024). By integrating genetic data with epidemiological models, researchers can trace the origins of outbreaks, identify high-risk transmission areas, and improve diagnostic tools. Applying forensic epidemiology aids in tracking zoonotic spillover incidents and strengthens One Health’s strategies for disease management.

### The investigation of brucellosis in the archeological record

Several methods can be used to investigate the evidence for brucellosis in the archeological record, including paleopathological, biomolecular, and modeling approaches (Bendrey et al., 2020). We will briefly describe the methodological context of these approaches and explore key evidence for the countries of the Middle East (Table 1 and Figure 2).

#### Paleopathology

Paleopathology is one method for studying diseases and conditions in ancient human and animal remains by examining skeletal or mummified remains, calcified soft coprolites, tissues, and historical documents. It involves the analysis of physical evidence to understand the health, diseases, and injuries that affected populations in the past (Waldron, 2020). The most common skeletal findings associated with brucellosis include infection of the bones, as indicated by osteomyelitis and periostitis, skeletal remodeling, and lytic lesions (Namiduru et al., 2004; Aydin et al., 2005; Arkun and Mete, 2011). Radiographs can reveal the presence of osteomyelitis and may also reveal information regarding the duration of the disease. Further, including microscopy can also support the establishment of dependable differential diagnoses for ancient diseases (Poinar and Cooper, 2000). A variety of studies have revealed the potential presence of brucellosis in an incomplete early human specimen through bone inflammation in the lumbar spine (D’Anastasio et al., 2011), vertebral lesions observed in the skeletons (Mutolo et al., 2012), and destructive lesions of the vertebral bodies and granulomatous tissue in lumbar vertebrae (D’Anastasio et al., 2009).

Paleopathology of brucellosis can help shed light on the history and evolution of the disease. It can provide insights into how it has spread and evolved. Paleopathology makes it possible to identify, connect, and characterize various aspects of human brucellosis. As an intracellular bacterium, any organ of the human body may be affected by *Brucella*, which could induce systemic infections by affecting the lymph and immune system, as well as by hematogenous spread to the sacroiliac and interphalangeal joints, spine, and knee (Turan et al., 2011). Skeletal manifestations of brucellosis are diverse and non-specific, with the potential for the presentation of many atypical forms, resulting in the under-recording of the disease in both present and past populations; also, diseases included in the differential diagnosis may differ, and depending on the age at which skeletal elements are affected (see review in Bendrey et al., 2020).

Table 1 summarizes some of the key sites with paleopathological results from the region. The earliest human osteoarchaeological evidence derives from an adult male skeleton from the Early Neolithic site of Ganj Dareh, Kermanshah province, Iran. It is associated with early evidence of goat husbandry (Merrett, 2004). This individual exhibits skeletal changes potentially indicative of an early stage of brucellosis infection, including new woven bone on the anterior and lateral surfaces of a thoracic vertebra and resorption of the superior anterior surface of a lumbar vertebral body, removing a portion of the annular ring. A range of probable brucellosis cases reported in the published literature comes from the Bronze Age Middle East (Table 1; Rashidi et al., 2001; D’Anastasio et al., 2009). One of the pioneer paleopathological reports of human brucellosis comes from Early Bronze Age Jericho, Palestine, which exhibited bone inflammation of the lumbar spine and pathological changes to both fibulae (Brothwell, 1965). Other Bronze Age archeological sites in Bahrain and Jordan also reported pathological signs of human brucellosis among human skeletal remains (Rashidi et al., 2001; Ortner and Frohlich, 2007). Paleopathological evidence is also derived from later periods, including Byzantine contexts at Khirbet es-Samra, Jordan (Table 1; Nabulsi et al., 2020), as well as medieval Mediterranean contexts beyond the Middle East (Mutolo et al., 2012).

#### Biomolecular detection of *Brucella* spp. from archeological samples

Detecting *Brucella* spp. in archeological human or animal skeletal samples can be challenging due to the potential degradation of DNA over time. For a consideration of the DNA damage encountered in ancient samples (see Dabney et al., 2013). Initial research employed PCR-based techniques to identify ancient diseases such as tuberculosis and leprosy caused by pathogenic *Mycobacteriaum* and plague due to *Yersinia pestis*; however, these methods encountered small DNA fragment lengths and non-specific amplification challenges. However, several measures can be employed to overcome these challenges. Distinguishing ancient DNA from contemporary contamination requires a fastidious approach and numerous validation procedures (Poinar and Cooper, 2000). Advanced quantitative PCR and probe-based techniques enhance pathogen detection in ancient skeletal remains (Marciniak and Poinar, 2019).

The utilization of ancient DNA has become more prevalent in examining pathogens that have undergone rapid or gradual evolution over extensive periods, ranging from hundreds to thousands of years ago. This approach allows for the investigation of ancient genomic diversity in a comparative manner, which has the potential to enhance our comprehension of the evolutionary changes that have occurred in pathogens over time (Dadar et al., 2025). The methods and techniques used to detect *Brucella* spp. in historical and old samples are comprised of PCR-based methods that are a powerful tool for detecting *Brucella* DNA in historical samples. Various PCR techniques can be employed, such as conventional PCR, real-time PCR, and nested PCR (Mutolo et al., 2012). Individual PCR approaches exhibit greater sensitivity than whole genome sequencing; hence, the latter is typically reserved for more robust PCR positives (Dabney et al., 2013). If *Brucella* DNA is present at sufficient quality, metagenomic sequencing can be employed to detect and identify the pathogen. Shotgun metagenomics can retrieve pathogen genomes from modern and ancient materials. For example, a calcified nodule from the skeleton of a 14th-century middle-aged adult male from Geridu, Sardinia, was subjected to shotgun metagenomic sequencing. This individual was found to be positive for *Brucella* DNA, and genome coverage was 6.5-fold. These genome’s sequence readings indicated DNA transition signatures (C to T and G to A) typical of ancient DNA (Dabney et al., 2013). Despite the limited coverage, single-nucleotide polymorphisms placed the medieval pathogen genome in a *B. melitensis* clade, including the well-studied Ether strain and two other recent Italian isolates (Kay et al., 2014). In addition, the utilization of proteomic analyses in examining ancient materials is now in its early phase of development, with only a limited number of findings documented related to ancient dietary samples (Greco et al., 2018). Biomolecular proteomic characterization of an ancient sample of Egyptian cheese showed that it was a dairy product obtained by mixing sheep/goat and cow milk (Greco et al., 2018). When working with archeological samples, it is helpful to employ multiple methods, as a combination of techniques can increase the chances of successful detection and validation. Furthermore, collaboration with experts in ancient DNA research and specialized laboratories is essential.

To date, there have been relatively few positive genetic analyses of archeological strains of *Brucella* spp. (Bendrey et al., 2020). In the Middle East, the earliest direct evidence for *Brucella* in the archeological record is reported by L’Hôte et al. (2024) who recovered a modest 3.45-fold coverage of a *B. melitensis* genome from a sheep petrous bone from Menteşe Höyük, Northwest Türkiye dated to 6,057–5,913 cal BCE, demonstrating that the pathogen was circulating in early livestock during the Neolithic period. From the Menteşe Höyük genomic evidence, L’Hôte et al. (2024) calculate the speciation time of *Brucella melitensis* and *Brucella abortus*, which they place at occurring some two millennia prior, during the earlier evolution of animal farming (L’Hôte et al., 2024).

Slightly further north of Türkiye is another early DNA record in the archeological record, this time of *Brucella abortus* (Sokolov et al., 2016). This study, initially undertaken on mitochondrial DNA from humans of the Early Bronze Age period in the North Caucasus, additionally showed *Brucella abortus* DNA recovered from one individual burial excavated from grave 1, kurgan 25 (settlement of Novosvobodnaya). Individuals in this study were radiocarbon-dated from 3,700 to 3,000 BCE (Sokolov et al., 2016), and the local animal husbandry practices were more focused on pigs and cattle (a finding in agreement with the understanding that *B. abortus* primarily infects cattle) (Sokolov et al., 2016).

There is also published evidence from Middle Bronze Age contexts at Gohar Tepe, Behshahr, Northern Iran (Kafil et al., 2014). The TaqMan real-time PCR method detected *Brucella* spp. in animal and human remains from this site. The target locus for this study was the DNA coding for a 31 kDa membrane protein (BCSP31). The extraction of DNA was performed from animal and human tooth samples (Kafil et al., 2014). The authors reported the presence of *Brucella* DNA for this marker in two young female skeletons and associated animal remains (Kafil et al., 2014).

Finally, the identification of a peptide sequence that may be associated with *Brucella melitensis* in the ancient Egyptian cheese sample mentioned above (Table 1) may represent the first direct biomolecular evidence for the role that the consumption of dairy products had in the past (Greco et al., 2018).

#### Paleoepidemiology, simulation modeling, and domestication

Paleoepidemiology is another scientific method that can be used to study the historical prevalence and impact of diseases in ancient populations. It is often assumed that the origins of farming and the increased contact between animals and humans considerably enhanced the risk of zoonotic brucellosis in the Middle East region during the Early Neolithic period (Moreno, 2014), i.e., from around 10,000 BCE. The domestication of sheep, goats, cattle, and pigs occurred in several locations in the Early Neolithic Middle East (Zeder, 2011). The changes in human-animal relationships from hunting animals for food to their domestication for food production significantly affected the evolution of zoonotic diseases (Diamond, 2002; Bendrey and Martin, 2021). To evaluate the impact of the transition to domestic animal husbandry from hunting on brucellosis dynamics, Fournié et al. (2017) modeled the dynamics of *Brucella melitensis* infection in Early Neolithic domestic goat herds in the Zagros Mountains using a stochastic and age-structured mathematical model simulating its spread within archeologically-defined settlement-based goat populations (Fournié et al., 2017). The study aimed to understand when, in the transition to goat herding, conditions were reached for the managed herds to sustain *Brucella* circulation permanently and, therefore, to be an endemic reservoir for human infection. The simulations indicated that brucellosis could have been present in relatively small domestic goat populations within the likely ranges estimated for these early farming settlements (Fournié et al., 2017). This resulted from the formation of dense domestic goat populations and significant changes in demographic characteristics, in that management decisions by early farmers changed the proportions of males and females in the herds—often preferentially retaining female goats into adulthood while selectively culling males. The modeling indicated that these management strategies would have inadvertently increased the transmission potential of the pathogen, thereby exposing the animals and humans to greater infection risk (Fournié et al., 2017). The recent genomic evidence from Menteşe Höyük, Northwest Türkiye, mentioned above (Table 1), supports this modeling work and confirms the presence of *Brucella melitensis* in Neolithic livestock in the region (L’Hôte et al., 2024).

**FIGURE 3:**
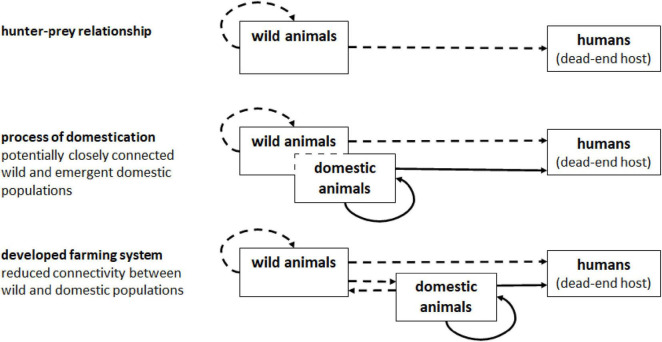
Schematic representation of *Brucella* epidemiology across animal domestication.

In the Zagros Mountains of Iran, the history of farming and the risk of brucellosis for human and animal communities has likely been a permanent risk for some 10,000 years (Abdi et al., 2020). It is perhaps notable that it is human–caprine relationships that still constitute the current primary challenges for zoonotic brucellosis epidemiology in these regions today (Bendrey and Fournié, 2021). The likely continuous circulation of *Brucella* spp. in the Middle East region may be indicated by the range of direct and indirect evidence reported (e.g., Table 1). Animal domestication did not create the first context for zoonotic infection; rather, it established the conditions for sustained zoonotic infection. Archeological evidence indicates that while infections can derive from hunting wild animals—e.g., vertebral brucellosis is reported in the partial skeleton of an *Australopithecus africanus* dated to the late Pliocene (Capasso, 2002; D’Anastasio et al., 2011). It was only with the domestication of livestock, e.g., goats, sheep, and cattle, that zoonotic brucellosis became endemic (Figure 3).

## Discussion

### The deep history of brucellosis in the Middle East

The changing relationships of humans and animals through time have had significant impacts on the incidence and ecology of infectious diseases (Bendrey and Martin, 2021). The epidemiology of these zoonotic infections was affected by different cultural, environmental, and biological factors. The study of the evolutionary history and current distribution of *Brucella* spp. can reveal important aspects of social dynamics, economic practices, and living conditions, as well as health implications of infectious diseases for past animals and humans. Brucellosis represents a perfect example of the important impact of zoonotic pathogens on human, wildlife, and domestic animal populations.

The dynamics of animal-human relationships as farming evolved created the conditions for disease emergence and endemicity. The close association of animals in large and dense herds with human communities led to increased opportunities for zoonotic infection for humans (Wolfe et al., 2007; Bendrey et al., 2020). A range of evidence points to the emergence of brucellosis as a zoonotic disease in the Early Neolithic Middle East with the origins of animal husbandry (Merrett, 2004; Moreno, 2014; Fournié et al., 2017; Moreno, 2021; L’Hôte et al., 2024). Evidence for human brucellosis has been also found across the wider region from the Bronze Age, for example in Bahrain, Jordan and the Persian Gulf (Brothwell, 1965; Rashidi et al., 2001; D’Anastasio and Capasso, 2017; Table 1 and Figure 2). In addition to zoonotic infections via direct contact, the consumption of infected dairy products was also likely a common infection route in prehistory. It is also the case that the most virulent *Brucella* strains were selected during the husbandry of animals (Moreno, 2014). Archeological evidence dating back to some 7,000 years ago in the eastern Mediterranean region of Anatolia points to early evidence for dairy consumption, representing a possible vehicle for brucellosis contagion (Evershed et al., 2008). In our study region, identifying a peptide sequence that may be associated with *Brucella melitensis* in the sample of pharaonic period cheese from Egypt provides insights into the presence of the latter in prehistory (Greco et al., 2018). Evidence for the dairy-related sources of human brucellosis has also been found from Roman Herculaneum, Italy, where SEM (scanning electron microscopy) analysis of carbonized cheese showed a coccoidal-like bacterium morphologically similar to *Brucella* (Capasso, 2002; D’Anastasio et al., 2009).

### The long-view perspective

This review demonstrates that since the origins of domestic farm animals, humans have been constructing and maintaining domestic animal populations in proximity to them that enable the emergence and maintenance of endemic zoonotic brucellosis. How we manage these animals and consume their products creates the routes whereby zoonotic transmission occurs. Brucellosis is thus a long-term and persistent health problem; it is not new. Although brucellosis sometimes only becomes “visible” with the emergence of microbiological testing, assumptions of recent emergence are likely misleading and do not recognize the significant potential for this disease to persist in farming systems. Knowledge of disease experience and expertise through the *longue durée* can make relevant and practical contributions to contemporary health knowledge and disease mitigation strategies. We argue here that the mobilization of this knowledge can make several contributions, as in the following examples.

(1) Understanding of time depth of disease presence and experience

This perhaps seems like a simple statement, but sometimes there is a tendency to assume that disease is only present when it becomes “visible.” For example, Amro et al. (2021) state that in Palestine, human brucellosis was introduced only recently and referenced the first human case reported in the West Bank in 1973. The widespread evidence of this disease spanning across time and space (Table 1 and Figure 2), and notably the identification of paleopathological evidence from Bronze Age skeletons in the Palestinian territories, attests to its antiquity in the region (Brothwell, 1965; D’Anastasio et al., 2011).

(2) Understanding of the evolution of pathogens and socio-ecological interactions

Historical records, genetic studies, and archeological evidence provide a means to trace the continuity of *Brucella* strains and their previous encounters with humans and animals. For some time, this bacterium has been attributed to the occurrence of brucellosis in diverse animal species. Its ability to endure and adapt has facilitated its interaction with humans over an extended period. Archeological discoveries have provided substantiating evidence of brucellosis in ancient human and animal remains.

The examination of *Brucella s*trains through genetic investigations can provide insights into the evolutionary trajectory of this bacterium (e.g., L’Hôte et al., 2024), elucidating how distinct strains have undergone adaptation to infect different host organisms successfully. Through the analysis of genetic markers present in contemporary and historical *Brucella* strains, researchers can gain valuable knowledge regarding the persistence of the pathogen and its historical interactions with various host species. The investigation of the persistence of *Brucella* strains and their historical contacts with humans and animals is currently a topic of active research. This line of inquiry offers significant insights into the past and present dynamics of brucellosis, contributing to our understanding of its history, epidemiology, and phylogeography. Archeological science has advanced rapidly in recent years, and significant discoveries will likely continue to emerge to improve our understanding of the long-term relationship between pathogen biological evolution and human cultural evolution.

(3) Understanding of the relationship between health knowledge and human action

In terms of improving long-term health knowledge to inform our decision-making and actions, as Woods writes (Woods, 2011), to understand historical health policy, it is helpful to examine “the context in which diseases emerged, were made visible, and were constructed as pressing problems in need of state-led solutions.” The present review aims to chart what is known about the long-term record of brucellosis in the Middle East region. Presenting the deep-time evidence for this disease may contribute to building a solid foundation to inform effective future decision-making. We argue that public health policy can benefit from a well-founded understanding of the broader history of a disease.

(4) Challenges in Vaccine Development and Brucellosis Control: A Long-Term Health Knowledge Perspective

Improving long-term health outcomes through brucellosis control requires the resolution of ongoing challenges such as underreporting, weak surveillance systems, shortage of resources, and socio-political barriers inhibiting sub-regional cooperation. An enormous number of cases go unrecognized based on poor public education, lack of easily accessible diagnosis units, and misattributing signs and symptoms, hence resulting in delayed therapy and more transmission. Early detection, case management, and reporting depend on initiatives for community health education and medical professionals improving their own. Furthermore, improving epidemiological tracking and response initiatives requires investment in cross-border data-sharing programs and border surveillance systems.

The history of diagnostic and vaccination approaches provides valuable lessons for improving long-term disease prevention and control. Traditionally, brucellosis vaccine programs utilized live attenuated vaccines (e.g., S19, RB51, and Rev-1), which were adequate but problematic from a human safety aspect. Advances in subunit and recombinant vaccines are hopeful options, though with issues regarding distribution, affordability, and acceptability. Raising awareness among cattle owners, vets, and politicians is key to improving take-up and vaccine compliance. Concurrent with these developments has been improvement in diagnostic reagents, ranging from serological tests to molecular diagnostic reagents. However, enhanced precision in detection is balanced by access and affordability limitations in guaranteeing their widespread application. Organizing training sessions between healthcare professionals and researchers will promote diagnostic speed and disease surveillance to guarantee long-term health intelligence and reinforce sustainable control and eradication of brucellosis.

## Conclusion

In conclusion, this review emphasizes the enduring nature of brucellosis as a long-term and persistent health issue, with roots as an endemic zoonosis extending back to the origins of domestic animal farming. The management practices surrounding these animals and the consumption of their products play crucial roles in facilitating zoonotic transmission. Contrary to assumptions of recent antiquity, evidence from archeological findings demonstrates the disease’s presence in human populations since the emergence of farming in the Early Neolithic.

Moreover, the study highlights the importance of leveraging knowledge from the long-term historical record of brucellosis to inform contemporary health knowledge and disease mitigation strategies. By understanding the time depth of disease presence, the evolution of pathogens, and the relationship between health knowledge and human interventions, significant contributions can be made to public health policy and decision-making processes. This comprehensive understanding provides insights into the challenges necessary for eradicating the disease. Ultimately, by integrating historical knowledge with contemporary approaches, we can develop more robust strategies to combat the persistent threat of brucellosis in farming systems and safeguard public health.

## References

[B1] Abdi M. Mirzaei R. Lohrasbi V. Zamani K. (2020). Zagros Mountains: A region in Iran with extremely high incidence of *Brucellosis*. *Infect. Control Hosp. Epidemiol.* 41 380–382. 10.1017/ice.2019.378 31971123

[B2] Abedi A.-S. Hashempour-Baltork F. Alizadeh A. M. Beikzadeh S. Hosseini H. Bashiry M. (2020). The prevalence of *Brucella* spp. in dairy products in the Middle East region: A systematic review and meta-analysis. *Acta Trop.* 202:105241. 10.1016/j.actatropica.2019.105241 31669529

[B3] Addis M. (2015). Public health and economic importance of brucellosis: A review. *Public Health* 5 68–84. 10.12691/ajeid-5-2-2

[B4] Akar K. Brangsch H. Jamil T. Yıldız G. Baklan E. A. Eroğlu B. (2024). “Genomic analysis of *Brucella* isolates from animals and humans, Türkiye, 2010 to 2020”. *Eurosurveillance* 38:2400105. 10.2807/1560-7917.ES.2024.29.38.2400105 39301739 PMC11484290

[B5] Alsulami Z. Conroy S. Choonara I. (2013). Medication errors in the middle East countries: A systematic review of the literature. *Eur. J. Clin. Pharmacol.* 69 995–1008. 10.1007/s00228-012-1435-y 23090705 PMC3621991

[B6] Amro A. Mansoor B. Hamarsheh O. Hjaija D. (2021). Recent trends in human brucellosis in the West Bank, Palestine. *Int. J. Infect. Dis.* 106, 308–313. 33864924 10.1016/j.ijid.2021.04.037

[B7] Anyanwu U. Wang Y. Walker A. Dimov A. Zinsstag J. Akladios Y. (2024). A mathematical model of animal-human Brucellosis transmission in Armenia: Implications for prevention and control. *CABI One Health* 3:1. 10.1079/cabionehealth.2024.0020

[B8] Arimi S. M. Koroti E. Kang’ethe E. K. Omore A. O. Mcdermott J. J. (2005). Risk of infection with *Brucella abortus* and *Escherichia coli* O157:H7 associated with marketing of unpasteurized milk in Kenya. *Acta Trop.* 96 1–8. 10.1016/j.actatropica.2005.05.012 16061190

[B9] Arkun R. Mete B. D. (2011). Musculoskeletal brucellosis. *Semin. Musculoskelet. Radiol.* 15 470–479. 10.1055/s-0031-1293493 22081282

[B10] Aydin M. Fuat Yapar A. Savas L. Reyhan M. Pourbagher A. Turunc T. Y. (2005). Scintigraphic findings in osteoarticular brucellosis. *Nucl. Med. Commun.* 26 639–647. 10.1097/01.mnm.0000167651.52724.68 15942485

[B11] Bagheri Nejad R. Krecek R. C. Khalaf O. H. Hailat N. Arenas-Gamboa A. M. (2020). Brucellosis in the Middle East: Current situation and a pathway forward. *PLoS Negl. Trop. Dis.* 14:e0008071. 10.1371/journal.pntd.0008071 32437346 PMC7241688

[B12] Bahmani N. Bahmani A. (2022). A review of brucellosis in the Middle East and control of animal brucellosis in an Iranian experience. *Rev. Res. Med. Microbiol.* 33 e63–e69. 10.1097/MRM.0000000000000266

[B13] Banai M. Itin R. Bardenstein S. (2018). Perspectives and outcomes of the activity of a reference laboratory for brucellosis. *Front. Vet. Sci.* 4:234. 10.3389/fvets.2017.00234 29354639 PMC5760530

[B14] Bendrey R. Fournié G. (2021). Zoonotic brucellosis from the long view: Can the past contribute to the present? *Infect. Control Hosp. Epidemiol.* 42 505–506. 10.1017/ice.2020.270 32618528

[B15] Bendrey R. Fournié G. (2023). How can deep time perspectives contribute to tackling contemporary One Health challenges, improving understanding and disease mitigation? *Res. Dir. One Health* 1:e9. 10.1017/one.2023.3

[B16] Bendrey R. Martin D. (2021). Zoonotic diseases: New directions in human–animal pathology. *Int. J. Osteoarchaeol.* 32 548–552. 10.1002/oa.2975 33821116 PMC8014110

[B17] Bendrey R. Cassidy J. P. Fournié G. Merrett D. C. Oakes R. H. Taylor G. M. (2020). Approaching ancient disease from a One Health perspective: Interdisciplinary review for the investigation of zoonotic brucellosis. *Int. J. Osteoarchaeol.* 30 99–108. 10.1002/oa.2837

[B18] Blasco J. M. Moreno E. Moriyón I. (2021). Brucellosis. *Vet. Vaccines* 12 295–316. 10.1002/9781119506287.ch22

[B19] Brangsch H. Sandalakis V. Babetsa M. Boukouvala E. Ntoula A. Makridaki E. (2023). Genotype diversity of brucellosis agents isolated from humans and animals in Greece based on whole-genome sequencing. *BMC Infect. Dis.* 23:529. 10.1186/s12879-023-08518-z 37580676 PMC10426126

[B20] Brothwell D. (1965). The paleopathology of early middle Bronze age remains from Jericho. *Jericho* 2 585–693.

[B21] Bruce S. D. (1887). Note on the discovery of a microorganism in Malta fever. *Practitioner* 39 161–170.

[B22] Capasso L. (2002). Bacteria in two-millennia-old cheese, and related epizoonoses in Roman populations. *J. Infect.* 45 122–127. 10.1053/jinf.2002.0996 12217720

[B23] Corbel M. Brinley Morgan W. (1975). Proposal for minimal standards for descriptions of new species and biotypes of the genus *Brucella*. *Int. J. Syst. Evol. Microbiol.* 25 83–89. 10.1099/00207713-25-1-83

[B24] Dabney J. Meyer M. Pääbo S. (2013). Ancient DNA damage. *Cold Spring Harb. Perspect. Biol.* 5:a012567. 10.1101/cshperspect.a012567 23729639 PMC3685887

[B25] Dadar M. Alamian S. Zowghi E. (2025). Comprehensive study on human brucellosis seroprevalence and Brucella species distribution in Iran (1970–2023). *Microb. Pathog.* 198:107137. 10.1016/j.micpath.2024.107137 39571831

[B26] Dadar M. Fakhri Y. Akar K. Ali S. Shahali Y. (2024). Human brucellosis and associated risk factors in the Middle East region: A comprehensive systematic review, meta-analysis, and meta-regression. *Heliyon* 10:e34324. 10.1016/j.heliyon.2024.e34324 39100474 PMC11296032

[B27] Dadar M. Shahali Y. Whatmore A. M. (2019). Human brucellosis caused by raw dairy products: A review on the occurrence, major risk factors and prevention. *Int. J. Food Microbiol.* 292 39–47. 10.1016/j.ijfoodmicro.2018.12.009 30572264

[B28] Dadar M. Shahali Y. Fakhri Y. Godfroid J. (2022). A comprehensive meta-analysis of Brucella infections in aquatic mammals. *Vet. Ital.* 58:28. 10.12834/VetIt.2427.14954.2 36586113

[B29] Dadar M. Tiwari R. Sharun K. Dhama K. (2021). Importance of brucellosis control programs of livestock on the improvement of one health. *Vet. Q.* 41 137–151. 10.1080/01652176.2021.1894501 33618618 PMC7946044

[B30] D’Anastasio R. Capasso L. (2017). “Microscopici segni paleopatologici,” in *il centro di microscopie dell’università degli studi dellaquila. 30 anni di attività*, eds Ragnelli A. M. Giammatteo M. Macchiarelli G. (Napoli: Idelson Gnocchi), 377–395.

[B31] D’Anastasio R. Staniscia T. Milia M. Manzoli L. Capasso L. (2011). Origin, evolution and paleoepidemiology of brucellosis. *Epidemiol. Infect.* 139 149–156. 10.1017/S095026881000097X 20447329

[B32] D’Anastasio R. Zipfel B. Moggi-Cecchi J. Stanyon R. Capasso L. (2009). Possible brucellosis in an early hominin skeleton from Sterkfontein, South Africa. *PLoS One* 4:e6439. 10.1371/journal.pone.0006439 19649274 PMC2713413

[B33] De Ley J. Mannheim W. Segers P. Lievens A. Denijn M. Vanhoucke M. (1987). Ribosomal ribonucleic acid cistron similarities and taxonomic neighborhood of Brucella and CDC group Vd. *Int. J. Syst. Evol. Microbiol.* 37 35–42. 10.1099/00207713-37-1-35

[B34] Diamond J. (2002). Evolution, consequences and future of plant and animal domestication. *Nature* 418 700–707. 10.1038/nature01019 12167878

[B35] Doganay M. Aygen B. (2003). Human brucellosis: An overview. *Int. J. Infect. Dis.* 7 173–182. 10.1016/S1201-9712(03)90049-X

[B36] El-Diasty M. El-Said R. Abdelkhalek A. (2021). Seroprevalence and molecular diagnosis of sheep brucellosis in Dakahlia governorate, Egypt. *Ger. J. Vet. Res.* 1 34–39. 10.51585/gjvr.2021.0006

[B37] Esmaeili H. (2014). Brucellosis in Islamic republic of Iran. *J. Med. Bacteriol.* 3 47–57.

[B38] Evershed R. P. Payne S. Sherratt A. G. Copley M. S. Coolidge J. Urem-Kotsu D. (2008). Earliest date for milk use in the Near East and southeastern Europe linked to cattle herding. *Nature* 455, 528–531. 18690215 10.1038/nature07180

[B39] Fournié G. Pfeiffer D. U. Bendrey R. (2017). Early animal farming and zoonotic disease dynamics: Modelling brucellosis transmission in Neolithic goat populations. *R. Soc. Open Sci.* 4:160943. 10.1098/rsos.160943 28386446 PMC5367282

[B40] Franco M. P. Mulder M. Gilman R. H. Smits H. L. (2007). Human brucellosis. *Lancet Infect. Dis.* 7 775–786. 10.1016/S1473-3099(07)70286-4 18045560

[B41] Godfroid J. Al Dahouk S. Pappas G. Roth F. Matope G. Muma J. (2013). A “One Health” surveillance and control of brucellosis in developing countries: Moving away from improvisation. *Comp. Immunol. Microbiol. Infect. Dis.* 36 241–248. 10.1016/j.cimid.2012.09.001 23044181

[B42] Grattarola C. Petrella A. Lucifora G. Di Francesco G. Di Nocera F. Pintore A. (2023). *Brucella ceti* infection in striped dolphins from Italian seas: Associated lesions and epidemiological data. *Pathogens* 12:1034. 10.3390/pathogens12081034 37623994 PMC10459742

[B43] Greco E. El-Aguizy O. Ali M. F. Foti S. Cunsolo V. Saletti R. (2018). Proteomic analyses on an ancient Egyptian cheese and biomolecular evidence of brucellosis. *Anal. Chem.* 90 9673–9676. 10.1021/acs.analchem.8b02535 30044608

[B44] Gwida M. Al Dahouk S. Melzer F. Rösler U. Neubauer H. Tomaso H. (2010). Brucellosis – regionally emerging zoonotic disease? *Croat. Med. J.* 51 289–295. 10.3325/cmj.2010.51.289 20718081 PMC2931433

[B45] Herrick J. A. Lederman R. J. Sullivan B. Powers J. H. Palmore T. N. (2014). *Brucella arteritis*: Clinical manifestations, treatment, and prognosis. *Lancet Infect. Dis.* 14 520–526. 10.1016/S1473-3099(13)70270-6 24480149 PMC4498663

[B46] Holzer K. Hoelzle L. E. Wareth G. (2023). Genetic comparison of *Brucella* spp. and *Ochrobactrum* spp. Erroneously included into the genus Brucella confirms separate genera. *German J. Vet. Res.* 3 31–37. 10.51585/gjvr.2023.1.0050

[B47] Holzer K. Wareth G. El-Diasty M. Abdel-Hamid N. H. Hamdy M. E. Moustafa S. A. (2022). Tracking the distribution, genetic diversity, and lineage of *Brucella melitensis* recovered from humans and animals in Egypt based on core-genome SNP analysis and in silico MLVA-16. *Transbound. Emerg. Dis.* 69 3952–3963. 10.1111/tbed.14768 36383491

[B48] Hotez P. J. Savioli L. Fenwick A. (2012). Neglected tropical diseases of the Middle East and North Africa: Review of their prevalence, distribution, and opportunities for control. *PLoS Negl. Trop. Dis.* 6:e1475. 10.1371/journal.pntd.0001475 22389729 PMC3289601

[B49] Hughes M. L. (1897). *Mediterranean, Malta or undulant fever.* London: Macmillan.

[B50] Ilyas M. Harpke M. Wareth G. (2024). Brucellosis in Iraq: A comprehensive overview of public health and agricultural challenges. *German J. Microbiol.* 4 10–20. 10.51585/gjm.2024.3.0039

[B51] Kafil H. S. Baha Hosseini S. Sohrabi M. Asgharzadeh M. (2014). Brucellosis: Presence of zoonosis infection 3 500 years ago in North of Iran. *Asian Pac. J. Trop. Dis.* 4 S684–S686. 10.1016/S2222-1808(14)60707-6

[B52] Kay G. L. Sergeant M. J. Giuffra V. Bandiera P. Milanese M. Bramanti B. (2014). Recovery of a medieval Brucella melitensis genome using shotgun metagenomics. *MBio* 5:e01337–14. 10.1128/mBio.01337-14 25028426 PMC4161259

[B53] Kousoulis A. A. Economopoulos K. P. Poulakou-Rebelakou E. Androutsos G. Tsiodras S. (2012). The plague of Thebes, a historical epidemic in Sophocles’ Oedipus Rex. *Emerg. Infect. Dis.* 18:153. 22261081 10.3201/eid1801.AD1801 PMC3310127

[B54] L’Hôte L. Light I. Mattiangeli V. Teasdale M. D. Halpin Á Gourichon L. (2024). An 8000 years old genome reveals the Neolithic origin of the zoonosis *Brucella melitensis*. *Nat. Commun.* 15:6132. 10.1038/s41467-024-50536-1 39033187 PMC11271283

[B55] Laine C. G. Johnson V. E. Scott H. Arenas-Gamboa A. M. (2023). Global Estimate of Human Brucellosis Incidence. *Emerg. Infect. Dis.* 29 1789–1797. 10.3201/eid2909.230052 37610167 PMC10461652

[B56] Liu Z. Wang C. Wei K. Zhao Z. Wang M. Li D. (2021). Investigation of genetic relatedness of *Brucella* strains in countries along the silk road. *Front. Vet. Sci.* 7:539444. 10.3389/fvets.2020.539444 33490123 PMC7817895

[B57] Mackie T. (1933). *Brucella* infections: With particular reference to undulant fever in this country. *Edinb. Med. J.* 40:T137. 29647042 PMC5306795

[B58] Marciniak S. Poinar H. N. (2019). Ancient pathogens through human history: A paleogenomic perspective. *Paleogenomics* 5 115–138. 10.1007/13836_2018_52

[B59] McDermott J. J. Arimi S. M. (2002). Brucellosis in sub-Saharan Africa: Epidemiology, control and impact. *Vet. Microbiol.* 90 111–134. 10.1016/S0378-1135(02)00249-3 12414138

[B60] McDermott J. Grace D. Zinsstag J. (2013). Economics of brucellosis impact and control in low-income countries. *Rev. Sci. Tech.* 32 249–261. 10.20506/rst.32.1.2197 23837382

[B61] Merrett D. C. (2002). Is pastoralism a pain in the . . . ? Palaeopathology in Early Neolithic Iran. American Association of Physical Anthropologists, Annual Meeting, Buffalo, New York. *Am. J. Phys. Anthropol.* S34, 112–113.

[B62] Merrett D. C. (2004). *Bioarchaeology in Early Neolithic Iran: Assessment of health status and subsistence strategy.* Winnipeg, MB: University of Manitoba.

[B63] Miller W. G. Adams L. G. Ficht T. A. Cheville N. F. Payeur J. P. Harley D. R. (1999). Brucella-induced abortions and infection in bottlenose dolphins (*Tursiops truncatus*). *J. Zoo Wildl. Med.* 65 100–110. 10367651

[B64] Moreno E. (2014). Retrospective and prospective perspectives on zoonotic brucellosis. *Front. Microbiol.* 5:213. 10.3389/fmicb.2014.00213 24860561 PMC4026726

[B65] Moreno E. (2021). The one hundred year journey of the genus Brucella (Meyer and Shaw 1920). *FEMS Microbiol. Rev.* 45:fuaa045. 10.1093/femsre/fuaa045 33016322

[B66] Moreno E. Blasco J.-M. Moriyón I. (2022). Facing the human and animal brucellosis conundrums: The forgotten lessons. *Microorganisms* 10:942. 10.3390/microorganisms10050942 35630386 PMC9144488

[B67] Moreno E. Middlebrook E. A. Altamirano-Silva P. Al Dahouk S. Araj G. F. Arce-Gorvel V. (2023). If you’re not confused, you’re not paying attention: *Ochrobactrum* is not *Brucella*. *J. Clin. Microbiol.* 61:e00438–23. 10.1128/jcm.00438-23 37395662 PMC10446859

[B68] Musallam I. Abo-Shehada M. Hegazy Y. Holt H. Guitian F. (2016). Systematic review of brucellosis in the Middle East: Disease frequency in ruminants and humans and risk factors for human infection. *Epidemiol. Infect.* 144 671–685. 10.1017/S0950268815002575 26508323

[B69] Mutolo M. J. Jenny L. L. Buszek A. R. Fenton T. W. Foran D. R. (2012). Osteological and molecular identification of brucellosis in ancient Butrint, Albania. *Am. J. Phys. Anthropol.* 147 254–263. 10.1002/ajpa.21643 22212927

[B70] Nabulsi A. J. Schönrock-Nabulsi P. Humbert J.-B. Desreumaux A. Wurst C. (2020). Intramural burials from the ancient byzantine settlement in khirbet es-Samrā in Jordan. *Int. J. Modern Anthropol.* 2 237–273. 10.4314/ijma.v2i14.2

[B71] Namiduru M. Karaoglan I. Gursoy S. Bayazıt N. Sirikci A. (2004). Brucellosis of the spine: Evaluation of the clinical, laboratory, and radiological findings of 14 patients. *Rheumatol. Int.* 24 125–129. 10.1007/s00296-003-0339-7 12811509

[B72] Nie L. F. Zhang F. Hu L. (2021). Nonlinear state-dependent pulse control for an SIRS epidemic model with varying size and its application to the transmission of brucellosis. *Math. Model. Nat. Phenom.* 16:58. 10.1051/mmnp/2021050

[B73] Ortner D. J. Frohlich B. (2007). The EB IA tombs and burials of Bâb edh-Dhrâ, Jordan: A bioarchaeological perspective on the people. *Int. J. Osteoarchaeol.* 17 358–368. 10.1002/oa.907

[B74] Pappas G. Memish Z. (2007). Brucellosis in the Middle East: A persistent medical, socioeconomic and political issue. *J. Chemother.* 19 243–248. 10.1179/joc.2007.19.3.243 17594917

[B75] Pappas G. Kiriaze I. J. Falagas M. E. (2008). Insights into infectious disease in the era of Hippocrates. *Int. J. Infect. Dis.* 12 347–350. 10.1016/j.ijid.2007.11.003 18178502

[B76] Poinar H. N. Cooper A. (2000). Ancient DNA: Do it right or not at all. *Science* 5482:416. 10.1126/science.289.5482.1139b 10970224

[B77] Rashidi J. Ortner D. Frohlich B. Jonsdottir B. (2001). Brucellosis in early bronze age jordan and bahrain: An analysis of possible cases of *Brucella spondylitits*. *Am. J. Phys. Anthropol.* 32 122–123.

[B78] Rayfield K. M. Mychajliw A. M. Singleton R. R. Sholts S. B. Hofman C. A. (2023). Uncovering the Holocene roots of contemporary disease-scapes: Bringing archaeology into One Health. *Proc. R. Soc. B* 290 20230525. 10.1098/rspb.2023.0525 38052246 PMC10697805

[B79] Sokolov A. Nedoluzhko A. Boulygina E. Tsygankova S. Sharko F. Gruzdeva N. (2016). Six complete mitochondrial genomes from Early Bronze Age humans in the North Caucasus. *J. Archaeol. Sci.* 73 138–144. 10.1016/j.jas.2016.07.017

[B80] Sun G. Q. Li M. T. Zhang J. Zhang W. Pei X. Jin Z. (2020). Transmission dynamics of brucellosis: Mathematical modelling and applications in China. *Comput. Struct. Biotechnol. J.* 18 3843–3860. 10.1016/j.csbj.2020.11.014 33335683 PMC7720096

[B81] Tadjbakhsh H. (2007). Sayyed Esma’il Jorjani, founder of Persian medicine. *J. Vet. Res.* 62, 131–140.

[B82] Turan H. Serefhanoglu K. Karadeli E. Togan T. Arslan H. (2011). Osteoarticular involvement among 202 brucellosis cases identified in Central Anatolia region of Turkey. *Intern. Med.* 50 421–428. 10.2169/internalmedicine.50.4700 21372451

[B83] Waldron T. (2020). *Palaeopathology.* Cambridge: Cambridge University Press, 10.1017/9781108583961

[B84] Wasserman N. (2012). Maškadum and other zoonotic diseases in medical and literary Akkadian sources. *Biblioth. Orient.* 69 426–436. 10.2143/BIOR.69.5.2967225 30588126

[B85] Wolfe N. D. Dunavan C. P. Diamond J. (2007). Origins of major human infectious diseases. *Nature* 447 279–283. 10.1038/nature05775 17507975 PMC7095142

[B86] Woods A. (2011). A historical synopsis of farm animal disease and public policy in twentieth century Britain. *Philos. Trans. R. Soc. B Biol. Sci.* 366 1943–1954. 10.1098/rstb.2010.0388 21624915 PMC3130385

[B87] Young E. J. (1995). An overview of human brucellosis. *Clin. Infect. Dis.* 21 283–289. 10.1093/clinids/21.2.283 8562733

[B88] Zeder M. A. (2011). The origins of agriculture in the Near East. *Curr. Anthropol.* 52, S221–S235.

